# Forensic Dermatology Is an Integral Subspecialty of Forensic Medicine

**DOI:** 10.7759/cureus.90269

**Published:** 2025-08-17

**Authors:** Philip R Cohen

**Affiliations:** 1 Dermatology, University of California, Davis Medical Center, Sacramento, USA; 2 Dermatology, Touro University California College of Osteopathic Medicine, Vallejo, USA; 3 Maples Center for Forensic Medicine, University of Florida College of Medicine, Gainesville, USA

**Keywords:** color, dermatology, forensic, human, medicine, pathology, report, skin, torture, trafficking

## Abstract

Forensic dermatology includes the evaluation of not only the skin and mucosa of a decedent but also that person's nails and hair. In addition to assessing decedents, forensic dermatology also encompasses the evaluation of victims of abuse, assault, deprivation, human trafficking, neglect, and torture. The dermatologist can contribute to the assessment of decedents; they can provide insight regarding the differentiation of both medication-associated reactions and benign melanocytic lesions from trauma to the skin or mucous membranes. Evaluation of nails for possible exposure to heavy metal poisoning can also be performed by the forensic dermatologist. In addition, an estimate of the time since death, the victim's body position when he or she died, and the possible cause of death can be determined when the dermatologist evaluates the decedent's lividity. Salient information can be obtained during the evaluation of tattoos by a forensic dermatologist; a unique tattoo, such as a mastectomy tattoo, or the presence of four or more random concordant tattoos, based on comparison to antemortem documentation, can possibly be used to establish a positive identification of the decedent. The pillars of forensic dermatology are collaboration, documentation, and education. A colorimetric scale for the forensic evaluation of decedents with skin of color has been established by the collaboration between a dermatologist and forensic pathologists. A template for a forensic dermatology expert analytical report, to communicate the observations made during a forensic dermatology consultation, has also been developed. Training in forensic dermatology for medical students and physicians is warranted. Subspecialties of forensic medicine traditionally include anthropology, ballistics, botany, entomology, and odontology. Forensic dermatology has recently been introduced to be a growing component of global forensic practice. In conclusion, forensic dermatology is an integral subspecialty of forensic medicine.

## Editorial

Introduction

Forensic medicine is also referred to as legal medicine. It usually involves the investigation of dead victims. Forensic medicine includes many subspecialties [1].

Forensic dermatology is one of the subspecialties of forensic medicine. It encompasses both the assessment of decedents and the evaluation of individuals who are the victims of human trafficking and torture [2-4]. It also includes the evaluation of not only benign skin lesions but also mucocutaneous drug reactions that can masquerade as traumatic injuries [5].

This editorial shall share some examples of the salient contributions provided by the forensic dermatologist. These shall include differentiating skin tumors and disorders from trauma-related injuries, describing an aspect of forensic onychology (such as the evaluation of nail changes that can be used to determine exposure to heavy metals), assessing the color of postmortem hypostasis that can provide insight into the cause of death, and establishing the positive identification of decedents by comparing their postmortem and documented antemortem tattoos. In addition, features regarding the pillars of forensic dermatology (including collaboration, documentation, and education) shall be summarized.

Subspecialties of forensic medicine

Forensic medicine involves applying medical knowledge to the investigation of a crime. Forensic medicine encompasses many areas such as anthropology, ballistics, botany, entomology, and odontology. In addition, forensic medicine also includes the subspecialties of both forensic dermatology and forensic pathology [1].

Definition of dermatology and forensic dermatology

Dermatology is derived from the Greek prefix "derma" meaning "skin" and the Greek suffix "logos" meaning "field of study" [6]. Hence, dermatology is the study of skin; indeed, it also includes the study of mucous membranes, nails, and hair. Forensic dermatology involves the legal aspects of mucocutaneous skin diseases and the dermatologic assessment of criminal investigations [7,8].

History of forensic dermatology

The subspecialty of forensic dermatology, to the best of my knowledge, was initially presented by Dr. Victor J. Rosen, the editor of the American Journal of Dermatopathology, in 1983; he published a paper demonstrating that dermatopathology had a significant role in forensic pathology [9]. During the next four decades, several investigators have emphasized the application of forensic dermatology in forensic medicine. Earlier this year, dermatologists from India have unveiled forensic dermatology in their country [10]. Indeed, forensic dermatology was recently introduced to be a worldwide subspecialty [11].

Examples of forensic dermatology

Forensic dermatology includes the identification of dermatologic diseases and skin infections on the skin of decedents. It also includes identification of primary cutaneous malignancies, manifestations of visceral cancers such as cutaneous metastases, and skin presentations of lymphoproliferative disorders such as lymphomas of the skin and leukemia cutis which may be associated with the cause of death of the individual. Some additional examples of the usefulness of forensic dermatology are differentiating dermatologic conditions or lesions from trauma, clues to the systemic exposure to a poison (such as a heavy metal) by evaluating the nails and hair, assessment of livor mortis, and the evaluation of tattoos [7,8].

Differentiate dermatological conditions or lesions from trauma

Dermatologists can aid in the differentiation of skin neoplasms or therapeutic interventions directed at the skin from blunt force trauma. Dermal melanocytosis includes a blue nevus, a nevus of Ito, or a nevus of Ota; these are benign pigmented lesions that can mimic trauma-induced bruises [12]. Similarly, some therapies such as coining or cupping can result in skin markings that mimic injuries caused by a blunt force to the skin [13,14].

Forensic dermatology can assist in distinguishing drug reactions from injury [5]. The sequela of prenatal exposure to thalidomide can present with healed deformities of the extremities that may be suspected to have originated from torture [15]. Also, the blue hyperpigmentation from hydroxychloroquine can masquerade as elder abuse [16].

Nails: forensic onychology can reveal exposure to a heavy metal

Forensic onychology is an aspect of forensic dermatology that involves the evaluation of fingernails and toenails to determine the etiology of systemic demise or the cause and manner of death. Systemic exposure to a heavy metal can result in visceral involvement and death. In addition to cutaneous changes, the victim's nails demonstrate morphologic changes compatible with exposure to the heavy metal [17].

Mees lines, one or more transverse bands of leukonychia of the nail plate, are traditionally associated with arsenic exposure. However, similar horizontal white bands can appear after exposure to either selenium or thallium. Other heavy metals, such as gold, lead, mercury, and silver, can also result in dyschromia of the nail plate. The heavy metals are deposited and can be detected in the nail plate to confirm the suspected diagnosis of exposure [18].

Livor mortis: a potential clue to the postmortem interval, the position at death, and the cause of death

Livor mortis is also referred to as dependent lividity or postmortem hypostasis. It develops on the gravity-dependent portion of the decedent. If the victim dies while lying on his or her back, the confluent redness of lividity will develop on the back. However, the hypostasis will not appear at locations where there is pressure against a bony structure, and this contact pallor will be evident over the shoulder blade regions of the upper back and often over localized areas of the buttocks [19-21].

Evaluation of livor mortis by the forensic dermatologist can be used to estimate the time since death. Postmortem hypostasis can also be used to evaluate the position of the body after death. And, by assessing the color of the lividity, it can occasionally provide a clue to the cause of death [19-22]. 

Postmortem Interval

The postmortem interval is an estimation of the time since death. Livor mortis becomes clinically apparent after only two hours. At this time, the red color can be blanched by applying pressure. However, between eight and 12 hours after death, the lividity becomes permanently fixed and cannot be blanched. Therefore, if the lividity can be blanched, the time since death is likely between two and eight hours. And, if the hypostasis is fixed, the estimated postmortem interval may be more than eight to 12 hours. Several factors can influence the rate of development of livor mortis; the most significant is the ambient temperature in which warm temperatures will accelerate the rate of lividity and cold temperatures will slow the development of hypostasis [19-21].

Body Position at Death

If the body of the decedent is moved prior to livor mortis becoming fixed, the distribution of the red lividity will change. However, once the hypostasis is fixed, the location of the red dependent lividity and the contact pallor will remain until the later stages of decomposition. If a victim is found in a prone position on their stomach and the lividity is present on their back, this would imply that the decedent had been repositioned from its original position at death [19-21].

Cause of Death

Livor mortis is typically red-purple. However, the skin develops a distinctive cherry-red to cherry-pink color when the cause of death is carbon monoxide poisoning; hypothermia, refrigeration, immersion in water, and certain poisons (cyanide and fluoroacetate) can also result in a similar color. In contrast, exposure to other poisons can result in livor mortis that presents with blue (aniline), brown (sodium nitrite), or green (hydrogen sulfide) skin color [19-22].

Tattoos

Tattoos can be used by the forensic dermatologist to aid in establishing a positive identification of the victim. They can also be helpful in discovering victims of human trafficking. In addition, certain tattoos (gunpowder tattoos or stippling) can aid in determining the range of fire of the gun used to kill a decedent [23-44].

Identification of the Victim

In a crime scene investigation, establishing the identification of the decedent is a primary objective. Comparison to the picture on a government-issued identification source is a manner of confirming a positive identification. Other methods to confirm a positive identification of the victim, which require prior documentation for comparison, are the evaluation of fingerprints or dental records or both [23,24].

Tattoos can be used to determine the identity of a victim. A unique tattoo can be the source of a positive identification [25-29]. In addition, like radiologic evidence, confirmation (with photographs or tattooist's records or medical documentation) of four or more individual tattoos at different body sites can be used to establish a positive identification [32,39].

A distinctive tattoo on the arm of a missing man was used to prompt the confirmation of the identification of that person. Jim Smith was a criminal and a police informant. He had been missing since April 7, 1935 [25-27]. 

On April 25, 1935, a large one-ton, 14-foot, recently captured tiger shark was on display in the Coogee Aquarium in Sydney, Australia. The shark regurgitated a bird, a cat, and a human left arm and hand; indeed, the disarticulated arm had been eaten by a smaller tiger shark that the larger shark had recently devoured. A distinctive tattoo of two boxers, facing each other with their fists poised for a fight, was observed on the forearm. The victim's brother and wife confirmed that Jim Smith had this distinctive tattoo. Law enforcement officers were able to evaluate the fingerprints on the hand; the fingerprints confirmed that the arm was from Jim Smith [25-27].

Certain medical tattoos, such as those applied post-mastectomy, can also be unique and enable a positive identification of a decedent. Many of the women who have breast cancer require surgical removal of one or both breasts as part of their management. Some of these women decide not to pursue surgical reconstruction of the breast or nipple/areola or both. In addition, some of the women also decline nipple-areolar-complex tattooing [28,29].

However, some of these breast cancer patients have elected to have a permanent mastectomy tattoo on one or both sides of their chests; these tattoos are often floral arrangements and are individually designed for each recipient. The tattoo artist often maintains a record of the completed tattoo, and it is likely that the patient also has photographic documentation of their tattoo. The tattoos provide emotional support and heightened self-esteem for these women and are so individualized that they can be used to establish a positive identification [28,29]. 

Radiologic evaluation of a decedent can be used to establish a positive identification [30,31]. Under these circumstances, antemortem medical or dental records of the sites are necessary for comparison to the postmortem roentgenograms [32,33]. These can include a unique bone abnormality or injury, or frontal sinuses, or dental X-rays [31,34-38].

Forensic anthropologists, pathologists, and radiologists have attempted to determine the number of concordant features, when comparing antemortem and postmortem radiographs of a decedent's bones, to establish a positive identification [39]. A uniform consensus, such as a specific number of concordant features that can be universally applied, has not been established [32,39].

Based on three standard radiographic views, a group of investigators established guidelines for the minimum number of concordant areas needed to confirm a positive identification. The number of areas depended on the bone being evaluated (Table 1) [39]. For example, while a 99% probability of correct identification would only require the existence of one concordant feature on the cervical vertebrae, a probability of 98% would necessitate four or more concordant features on either a lumbar vertebra or a thoracic vertebra [39].

**Table 1:** Probability of a positive identification associated with body area and number of concordant features on the bone

Body area	Number of concordant features	Probability of positive identification	Reference
Cervical vertebrae	≥1	99%	[39]
Femoral neck	≥1	97%	[39]
Femoral head	≥1	94%	[39]
Lateral cranium	≥2	97%	[39]
Lumbar vertebrae	≥4	98%	[39]
Thoracic vertebrae	≥4	98%	[39]

Antemortem documentation of a single unique tattoo, such as a mastectomy tattoo, would be sufficient to establish a positive identification of a decedent [28,29]. However, evaluation of individual non-distinctive tattoos in a decedent to provide a positive identification could be approached in a similar manner to the detection of concordant radiologic features. Therefore, based on the requirement of four or more concordant antemortem and postmortem features on the roentgenograms of either a lumbar vertebra or a thoracic vertebra to determine a positive identification with a 98% probability, a conservative recommendation to establish a positive identification using random individual tattoos on a decedent would require four concordant tattoos that had antemortem documentation [32,39].

Victims of Human Trafficking

Tattoos are used by human traffickers to exert control over their victims. The tattoos result in the stigmatization and dehumanization of the victim. The tattoos are frequently located on body sites that cannot be concealed, such as the neck [40,41].

Most of the tattoos created by human traffickers are homemade. Their quality is poor, their shading is inconsistent, and their lines are uneven. The appearance of tattoos on victims of human trafficking includes dollar signs, barcodes, crowns, and guns [40,41].

In addition to clinicians in an emergency room, other healthcare providers may have the opportunity to examine the skin of human trafficking victims during a medical encounter. Victims of human trafficking can present to a physician or healthcare worker for various issues including trauma, infections, and other healthcare needs. Trafficking-associated tattoos can be either prominently displayed or discovered on a complete skin examination of the victim by a dermatologist [41,42].

Stippling Associated With a Gunshot Wound

Identifying entrance, exit, and graze wounds is the first major external determination of a gunshot wound. Determining the range of fire is the second. The forensic dermatologist can play a role in both evaluations [43].

Close-range gunshot wounds include contact wounds, near-contact wounds, and intermediate-range wounds [43]. Stippling, also referred to as powder tattoo, is pathognomonic of an intermediate-range wound; this occurs when the muzzle is less than two feet from the victim [43]. Gunpowder stippling occurs when tiny unburned, partially burned, or burning grains of gunpowder create a punctate abrasion of the skin surrounding the entrance wound [43,44].

Pillars of forensic dermatology

Collaboration, documentation, and education are the pillars of forensic dermatology [45-47]. A collaboration between a dermatologist and forensic pathologists has created a colorimetric scale for the forensic evaluation of decedents with skin of color [45]. Like other subspecialties of forensic medicine, a template for the expert analytical report has been developed to be used when a forensic dermatology consultation has been performed [46]. Additional training in forensic dermatology for medical students, dermatology residents, and dermatologists is warranted [10,34,47].

Collaboration: Colorimetric Scale for Skin of Color

The colorimetric scale for skin of color is a practical classification scale for the clinical assessment, dermatology management, and forensic evaluation of individuals with skin of color. In contrast to the Fitzpatrick classification which classifies individuals as either white, brown, or black (of which the former and latter are not colors), the colorimetric scale is only used to assess people with skin of color and ranges from very light beige (skin color type 1) to very dark brown (skin color type 5). It is a simple scale to use to categorize persons with skin of color, and the evaluation can be rapidly performed; the classification is not based on race or ethnicity. Three forensic pathologists collaborated with a dermatologist to develop the scale so that the classification system fulfilled the pathologist's forensic investigative needs while concurrently providing the dermatologist with a system for assessing the management of dermatology patients [45].

Documentation: Forensic Dermatology Expert Analytical Report

When a consultant subspecialist aids in the crime investigation of a decedent, the subspecialist communicates the findings with the police, detective, crime scene investigator, forensic pathologist, and attorneys by preparing a forensic expert analytical report. Templates have been developed that can be used by anthropologists and entomologists to present their findings in a uniform manner. A similar template had been developed for dermatologists who evaluate a decedent as part of the investigation of that individual's death. The dermatology expert analytical report is a succinct document that enables the evaluating dermatologist to present all the pertinent findings in an organized manner so that the content of the report can be readily understood and used by the other people involved in the investigation of the crime and/or the litigation of the suspected assailant [46].

Education and Training in Forensic Dermatology

The Department of Dermatology at the University of California, San Francisco, implemented a pilot forensic dermatology curriculum to train 14 dermatology residents in September 2021. The residents virtually received a one-hour didactic session. The investigators noted that the dermatology residents had an improvement in their ability to describe skin findings of torture or abuse and that their attitudes towards participating in the evaluation and care of patients with these issues also improved [34].

In a multicenter follow-up study from October 2023 through April 2024 of seven dermatology residency programs, dermatology residents received a one-hour virtual lecture. The emphasis of the teaching was on the cutaneous manifestations of traumatic injuries that may occur in refugees and asylum seekers. The researchers had noted that training programs in forensic dermatology were limited in medical schools and residency programs; they suggested that longitudinal learning of forensic dermatology beyond residency should be assessed and promoted [47].

Like the United States, only the clinical management of skin conditions is emphasized in the dermatology training curriculum in India; there is only a minimal focus on the medicolegal implications of forensic dermatology. Students, residents, and practicing dermatologists do not receive adequate mentoring in recognizing the cutaneous and mucosal signs of abuse, factitious disorders, self-inflicted injuries, and torture. The investigators from India proposed that to enable physicians to effectively manage patients with these conditions, forensic dermatology must be integrated into the teaching curriculum [10]. 

Conclusion

Forensic dermatology is an essential component in the assessment of a decedent and the investigation of victims of human trafficking and torture. The role of the dermatologist in evaluating decedents includes differentiating trauma to the skin or mucous membranes from either benign melanocytic lesions or reactions caused by medications. It also includes the evaluation of nails for possible exposure to heavy metal poisoning. In addition, the dermatologist can aid in the assessment of livor mortis to provide an estimate of the postmortem interval, the position of the victim when he or she died, and the possible cause of death. Also, evaluation of tattoos can potentially provide salient information to establish a positive identification of the decedent based on either the presence of a unique tattoo such as a mastectomy tattoo or the presence of four or more random concordant tattoos based on comparison to antemortem documentation. The pillars of forensic dermatology are collaboration with other individuals involved in the investigation of a decedent such as the forensic pathologist, documentation of the skin, hair, nail, and mucous membrane findings, and education of clinicians regarding the mucocutaneous stigmata of forensic dermatology-related findings. In conclusion, forensic dermatology is an integral subspecialty of forensic medicine.
